# Differences in thinking flexibility between novices and experts based on eye tracking

**DOI:** 10.1371/journal.pone.0269363

**Published:** 2022-06-30

**Authors:** Mengyao Zhu, Defu Bao, Yuxiang Yu, Danni Shen, Minzhe Yi

**Affiliations:** Art and Design Institute, Zhejiang Sci-Tech University, Hangzhou, Zhejiang, China; University of Salento, ITALY

## Abstract

The influence of thinking flexibility on design is often underestimated by researchers in the field of design education. In this study, morphological analysis was used as a tool to develop design proposals and eye tracking technology was applied to track the attention. The feature of thinking activities in problem-solving between two groups (novice and expert) was analyzed by Heat map and Gaze plot in qualitative, and measured by indicators such as fixation and saccade in quantitative. Findings suggested that, i) Experts showed more fixation duration and fixation numbers in thinking activities, and the attention containing more AOIs was positively related to the rationality of the scheme. ii) Saccades with greater amplitude were more beneficial to the novelty of scheme. iii) Experts considered the information of each block in a balanced way, while novices tended to ignore unimportant blocks. These results will have a far-reaching impact on the development of designers’ thinking and help novices to exercise creative thinking and produce high-quality designs.

## Introduction

Thinking flexibility is an important but yet sub-optimally understood design strategy. It has a positive impact on stimulating creative strategies and creative skills of students in interdisciplinary education. An interesting creative process is produced by a series of complicated thoughts, it is crucial to be flexible in these complex creative problem-solving [1]. Besides, compared with conservative thinking, flexible thinking can be more conductive to produce high-quality designs. Shen had proved that flexibly thinking objects in different perspective directly improves the type, quantity and completeness of students’ design activities [2]. Therefore, it is necessary for educators to teach students appropriate flexible thinking to improve their design skills.

However, the features of thinking activities on the designers with different expertise levels have obvious difference. Experts can select and apply their thinking modes more flexibly, but novices have limited ability to do so because of the lack of experiences [3]. Therefore, a requirement to understand individual differences of thinking flexibility between experts and novices has been raised for education. It can form a foundation for developing personalized education of thinking way, which can help improve the design skills of each student [4]. In particular, more researches are needed to analyze the directional difference of thinking flexibility and its impact on enhancing creative process [5]. Some important issues remain unexplored, such as visualization of abstract thinking process, evaluation of multiple features of thinking.

In previous studies, some researchers tend to use concept map and linkogragh to explore the relationship between behaviors to understand the design thinking process [5]. And several studies of design thinking depend on protocol analysis to record and then analyze what designers say and what they are doing. Although some studies may acquire interesting findings, these traditional methods just made a simple information processing of the design thinking [6], which are difficult to record the potential feature of thinking activity objectively and accurately. With the development of technology, more trending technologies were used to understand people’s natural thinking. Eye tracking technology, which is an objective and innovative method of behavior recording, has been highly valued by enterprises and research institutions because of its advantages in reflecting people’s behavior intention with physiological data [7]. Previous researches mostly discuss participants’ eye movement when they look at static pictures [8]. However, there is little research on exploring eye movement during a dynamic design process.

To address above research gap, this study focused on how designers distribute their attention when they are thinking solutions in flexible thinking modes. It aims to explore significant features of thinking flexibility among designers with different expertise levels and its relationship with the design results. Results can promote the quality of design by informing novice on how to training flexible design thinking, and accelerate the improvement and supplement of design education.

## Literature review

### Design thinking

#### Creative design thinking

Over the past decades, design thinking (DT) has attracted increased interest from researchers. Studies on DT have been conducted by researchers not only in fields of design but also in education [9]. In the field of design research, DT was always used to explain the way designers think and work [10]. Whereas in the field of education, researchers believed creative design thinking can promote students’ better cooperation [11] and enhances their interest in exploring solutions [12], which can help designers to promote design behavior and design results. Therefore, creative design thinking has been regarded as the key content for researchers to analyze design thinking.

Recent studies have demonstrated that creative design thinking is usually related to concept combination and thinking flexibility [13]. Concept combination refers to combining two or more concepts to create a new concept. It can effectively enhance the innovation of design due to the diversity of objects and the multi-solution of problems [14, 15]. In addition, Mobley et al. found that products composed of different concepts have higher novelty than those combined with concepts of similar categories [16].

Thinking flexibility is also related to creativity, which refers to the ability to recognize objects in different thinking modes. It has been proven that creative ideas can be facilitated when designers flexibly apply different thinking modes [17]. Martinec et al. found that experienced designers tend to move more flexibly between different sub-goals to seek new opportunities when solving complex problems [18]. In recent years, researcher has found that we need to pay attention to exploring the general features when designers thinking in different thinking modes [5], which has been regarded as one sign of creative thinking during the problem-solving process [19].

#### Differences of design thinking among novices and experts

Obviously, designers with different expertise levels have great differences in design thinking, attention performance and so on [20]. In order to explore it, Scholars previously compared problem-solving process and design activity of designers.

Since experts have more experience knowledges, they can adopt different thinking modes flexibly between problem and solutions [3], but novices have limited ability to do so. Furthermore, researchers found that experts and novices have different organizational abilities for cognitive knowledge [21]. This idea has also been endorsed by Mintzes, who believed that experts could usually make the previous knowledge form a strong hierarchical framework, which allows them to reuse these concepts in more fields [22]. In addition, previous research indicated that novices usually use vertical conversion to explore a more detailed solution in depth, while experts usually use horizontal conversion, that is, the conversion from one area to a slightly different area, to expand the problem space and explore some core ideas [23].

#### Morphological analysis

Morphological analysis method was put forward by Dr. Fritz Zwicky in the 1940s, which is an effective method to exercise design thinking. Its basic principle is to decompose the problem into several independent factors according to its components or functional units, then take each factor as an independent variable and find out its corresponding solutions, use tables to arrange and combine solutions to get the final scheme. In the field of design, morphological analysis can break through the limitations of thinking by forced combination, and come up with some counter-intuitive solution, so as to comprehensively improve the innovation of proposals.

Previous studies found that using morphological analysis to consider different factors is helpful for designers to broaden and deepen the initial concept and to create a large number of different alternatives [24]. Besides, compared with concepts created by other tool such as brainstorming, designers were more likely to select these broaden and deepen concepts as their most creative, unique and favorite designs [25]. Therefore, morphological analysis was used as a tool to develop design proposals in this study.

### Eye tracking

#### Eye tracking uses in design

With the development of technology, more trending technologies were used to understand people’s natural thinking. For example, some researchers used electroencephalogram (EEG) to detect potential thinking activities, which is a direct method to measure cognitive processes and sensitive to the instantaneous changes in the brain [26]. And other technologies, such as electrodermal activity (EDA), electrocardiography (ECG), have also been employed in the relevant attention-related literature.

However, the thinking activity is largely an unconscious phenomenon that the user tends to look at where he or she would like to move before the actual movement [27]. And it can be externally manifested as visual behavior and usually expressed by attention movement. Therefore, eye tracking technology is considered the most adequate methodology to explore users’ thinking activities in this study [28]. Besides, eye movement data is quicker and more directly collected than much other cognitive data [29]. It can reflect attention change in real time and is suitable to act as an objective indicator to study abstract concept in complex internal process [30]. For example, Keskin used a variety of eye movement data to explore the influence of the independent variables, such as task difficulty and expertise level on the cognitive strategies of map users [21]. Debue detected attention through gaze movements, to measure the factors that generate mental workload [31].

#### Meaning of eye movement metrics

According to previous study, the performance of thinking activities can be extracted by using both fixation and saccade related eye tracking metrics. On the one hand, fixations are stable point during a certain time span (at least 80 to 100 ms), and indicate the users’ content interpretation at that location. For instance, higher fixation duration and fixation numbers indicate that the content is attractive [32]. Jacob also pointed out that the number of fixations on each area of interest can be a measure of the importance of the information content [27]. On the other hand, saccades are short (typically 30–80 ms) and voluntary. Eye movements between saccade and two fixation points can be visualized as scan paths. It can be used to know the order individuals moved from one area to another area. For example, Goldberg et al. used the metrics like fixation duration and saccade to sum up the browse order and discuss its potential influence on targeted search while individuals navigated a web page [33].

### Evaluation of design schemes

Scheme design is the initial stage of conceptual design, and how to choose the best one is the key problem that must be solved. Cognitive psychologists consider novelty and fluency as the primary measures of ability to generate ideas [34]. However, this is not quite adequate for engineering design. Sarkar suggested that rationality is also a factor that has a direct impact on creativity [35].

Novelty is a standard to measure how unusual or unexpected an idea is, Shah et al. put forward a method to measure novelty: firstly, the product should be decomposed into different components based on its key functions or characteristics, and then novelty in concept or implementation level is analyzed by comparing with previous products [36].

Rationality can measure the feasibility of an idea and how close it is to the design specification. It is an independent measure related to physical properties such as performance. Shah also insisted that the ultimate goal of engineering design was to create a better product. If there was no feasible in physics, all design efforts would be in vain. Therefore, evaluation of rationality must be included in the overall evaluation of scheme [36].

Quantity is the total number of ideas generated by individuals during a designated amount of time. Many people thought that generating more ideas will increase the chances of producing better ideas [37]. As such, psychology often uses fluency (quantity) to measure a person’s creativity [34].

At last, we identified three types of outcome based metrics: novelty, rationality and quantity.

## Methods

### Tasks and stimulations

The task of this study is to design a portable multifunctional thermos cup using morphological analysis in 45 minutes. The “Think aloud” method was used to collect cognitive data while eye tracking equipment was used to capture their eye movements. The materials for experiment included a concept handout, a presentation with design case using morphological analysis, a design task statement, a practice task and a A3-sized morphological analysis card. The morphological analysis card is presented in the form of a table with 6 rows by 8 columns, including 6 sub-functions: foldable and supported cup, ways of drinking, portable belt, cup cover, heat preservation method and environmental protection. What is different from the traditional table is that we classify sub-function into three categories with different importance: key, important and secondary. We distinguish them by several colors, but in order to avoid the potential guidance, there is no color distinction on the given table (see Table 1 for details).

**Table 1:** Experimental information.

participants	24 participants	
	12 undergraduate students (5 males and 7 females, average age: 20)	
	12 professors (3 males and 9 females, average age: 42.75, average tenure: 12.9 years)	
	Average visual acuity: 1.1	
Stimulation (morphological analysis table)	Three sub-function blocks of different importance are arranged out of order: each block contains two specific sub-functions.	
	Block 1 (Important)	Sub-function 1: Portable belt
	Block 1 (Important)	Sub-function 2: Cup cover
	Block 2 (key)	Sub-function 3: Cup that can be folded and supported
	Block 2 (key)	Sub-function 4: Drinking water
	Block 3 (secondary)	Sub-function 5: Heat preservation mode
	Block 3 (secondary)	Sub-function 6: Environmental protection
Independent variables	2 expertise levels (experts vs. novices)	
Dependent variables	eye tracking metrics: fixation duration, the number of fixation, the number of saccade	

### Participants

Undergraduate students and professors in the College of Art and Design of Zhejiang Sci-Tech University participated in the study. They are all from design majors, none of them had specific experience with morphological analysis before. Students are aged between 18 and 21 and professors are aged between 34 and 52. For more information, please refer to Table 1. Participants were screened for normal (or corrected to normal) vision acuity, and for no history of migraine headaches or epilepsy. Those with bifocals and contact lenses were not further considered due to limitations of eye tracker. All were familiar with the conceptual process and had experience in designing sketches. All the participants provided written consent for publication of raw data and were treated in accordance with national and international norms governing the use of human research participants. The Art and Design Institute of Zhejiang Sci-Tech University granted permission for the performance of the experiment.

### Equipment

**Fig 1:**
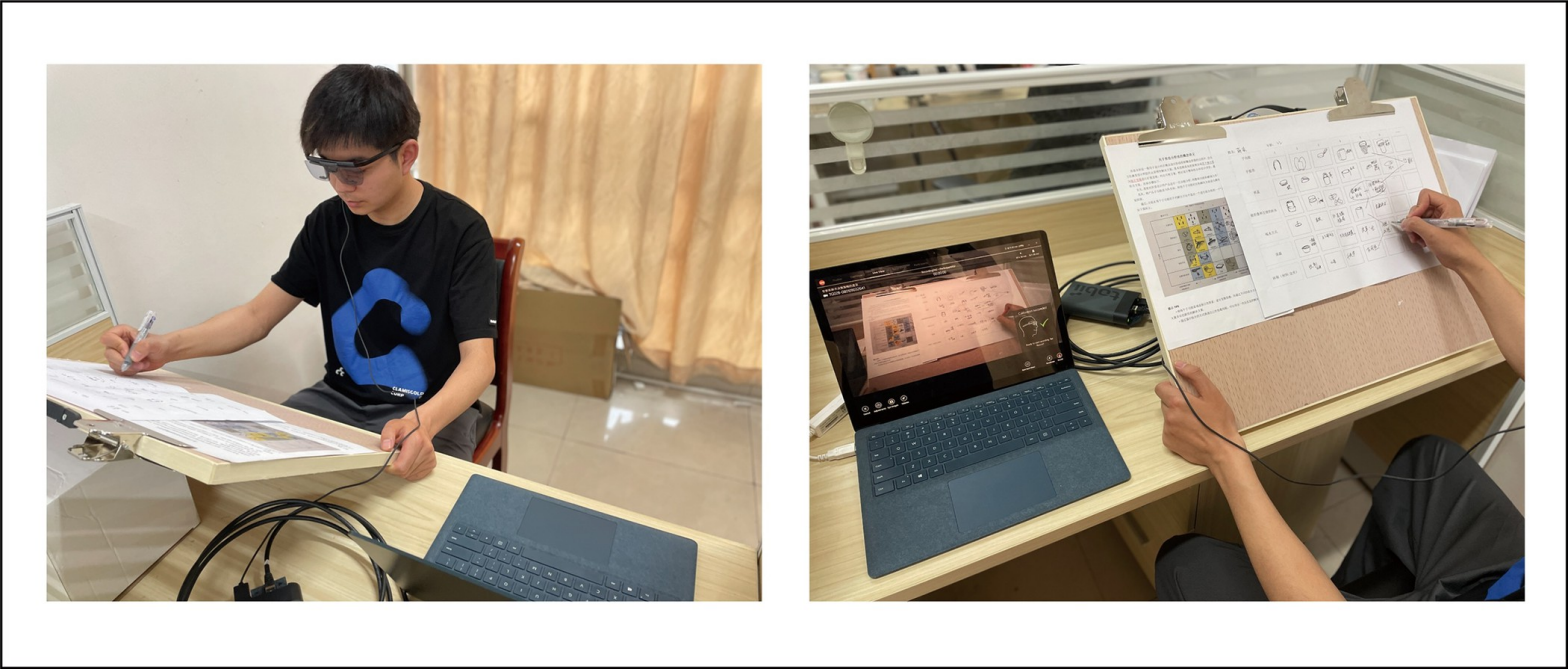
Experiment setting.

The study was conducted in a laboratory, a closed space without direct light, in Zhejiang Sci-Tech University. The test room contained an eye tracking equipment, chairs, tables, a drawing board and a recorder. Adjustments were made to maintain the participants’ eyes at 30cm from the drawing board. An electronic recorder captured all comments in the process by participants. Eye movement were collected using Tobbi Pro Glasses, which was recorded at the sampling frequency of 250 Hz. The Tobbi Pro online analysis software was responsible for processing metrics like fixations and saccades. In addition, the mobile phone should be kept away from it, to reduce the interference of the magnetic field on the equipment and the psychological dependence of the subjects on the network information. Fig 1 shows the experiment set up.

### Procedure

Objective: record the eye movements of participants’ thinking in the design process, and analyze the process qualitatively and quantitatively to find out the potential features of thinking activities.

**Fig 2:**
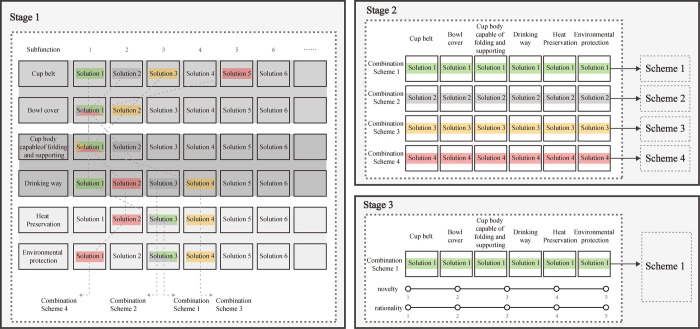
Process of combination scheme.

Steps:

1. the concept handout of morphological analysis including a design case was showed, the design task and notes in using equipment were explained.
2. After reading the handout carefully, participants were asked to do a practice task. They were required to use a table with 3 rows by 4 columns to design a bicycle until they can use morphological analysis correctly.
3. Then, the eye tracker headband was mounted in the help of researchers, followed by a short calibration procedure. Adjustments were made to maintain the participants’ sightline perpendicular and at 30cm from the drawing board.
4. Participants were required to design a portable multifunctional folding thermos cup, with a think-aloud protocol about when they persist in the same area and when they are flexible on objectives in different thinking modes. The corresponding solutions of each element should be filled in each listed sub-function (participants can use various formal and informal symbols, such as graphics, words and so on). In addition, they were allowed to complete task at their own pace that don’t have to follow the prescribed order, as long as the task was completed within 45 minutes.
5. After completing the table, participants should select a solution from each sub-function line to combine into a whole scheme, and draw a sketch of the combined scheme on the draft paper. The process was shown in Fig 2.

### Data processing

#### Create time of interest (TOI)

Videos of each participant were analyzed in tobbi pro lab software. In order to find out the time segment when designers flexibly act in different thinking modes, two set of coding schemes were applied in this research. The first one is based on participants’ eye-tracking data. The second coding scheme is based on participants’ language, which collected from think-aloud protocol.

**Fig 3:**
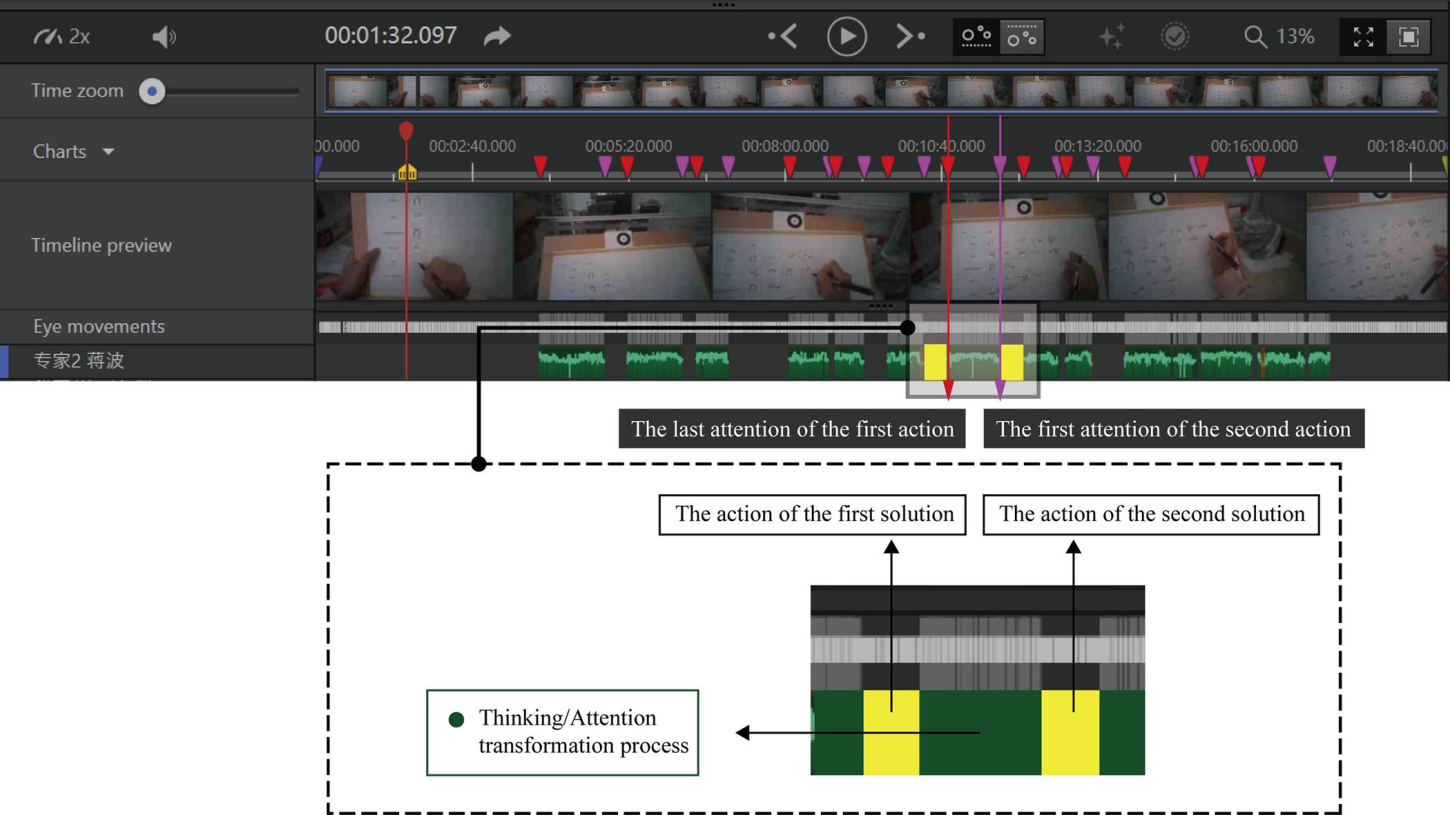
TOI (time of interest) division.

Firstly, all segments about the thinking activities between two solutions were sort out, in the way of marking the last attention of one action (solution) and the first attention of the next action (solution). As shown in Fig 3. We encoded these segments in the name of TOI1, TOI2, TOI3 etc. Second, the key sentences such as “now I want to find inspiration from the solutions of other sub-functions” “I’ve been focusing on the handbag solution. Now I’m going to spread my mind and look at other solutions.” were selected in think-aloud protocol, which indicate there are two different thinking modes in this process. We encoded these sentences as S1, S2, S3 etc, and exclude TOIs with no sentences existing. Table 2 shows the general results of the segmentation. Finally, in order to control variables, 12 valid TOIs (time of interest) from each video were selected and 240(12*20) pieces of valid data were obtained.

**Table 2:** General results of segmentations.

	Total segments (sentences)	Total segments (eye tracking)	Total design time (min)
Novice	140	216	387
Expert	154	204	492

The corresponding morphological analysis table were then added to complete the automatic matching of the gaze points. The quality of the automatic matching points should be checked, and adjustment were made if there is any difference between the gaze points in the video and those in the pictures.

#### Visual data analysis

**Fig 4:**
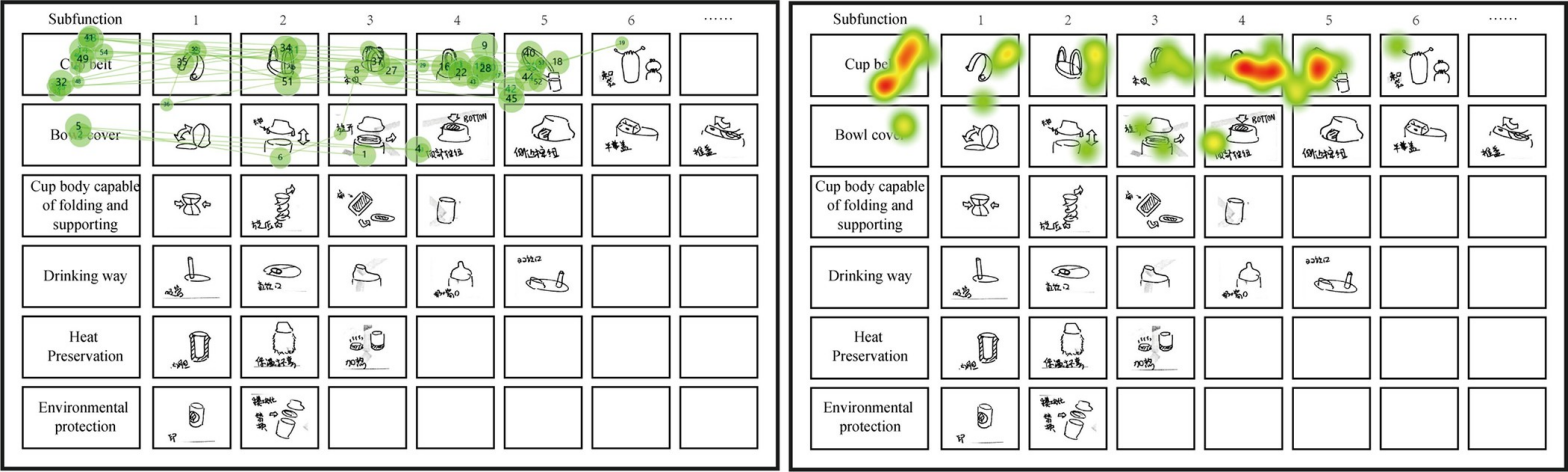
A novice case of gaze plot and heat map.

The visualization graph includes gaze plot and heat map. The size of point in gaze plot is determined by the duration of fixation it represents. A long fixation is represented by a large point and a short fixation by a small point. Heat Map uses different colors to illustrate the number of fixations participants made within certain areas of the stimulus or for how long they fixated within that area. Red usually indicates the highest number of fixations or the longest time fixating there, and green the least. As shown in Fig 4.

**Fig 5:**
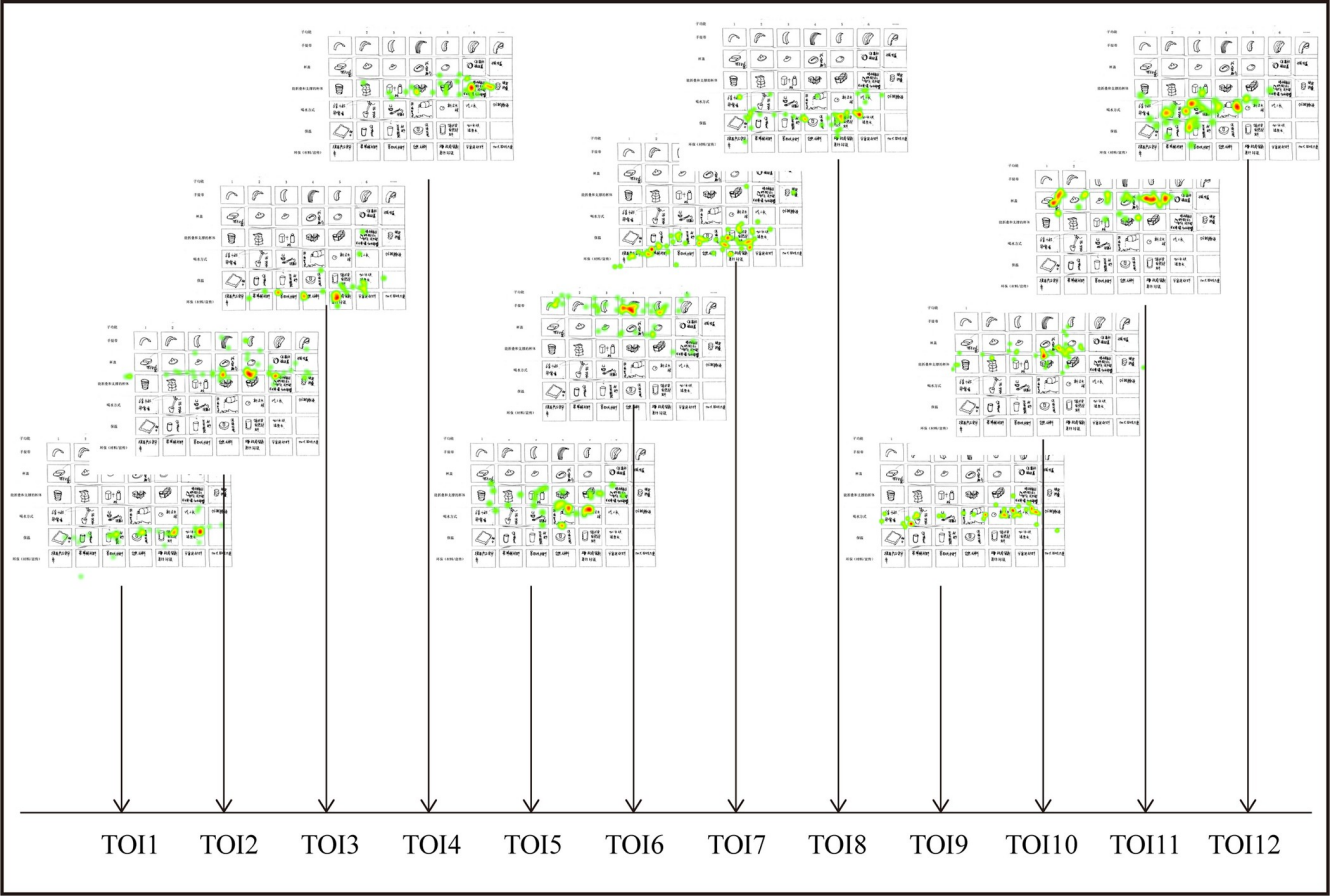
Heatmaps of the eye-tracking during 12 TOIs (a novice).

**Fig 6:**
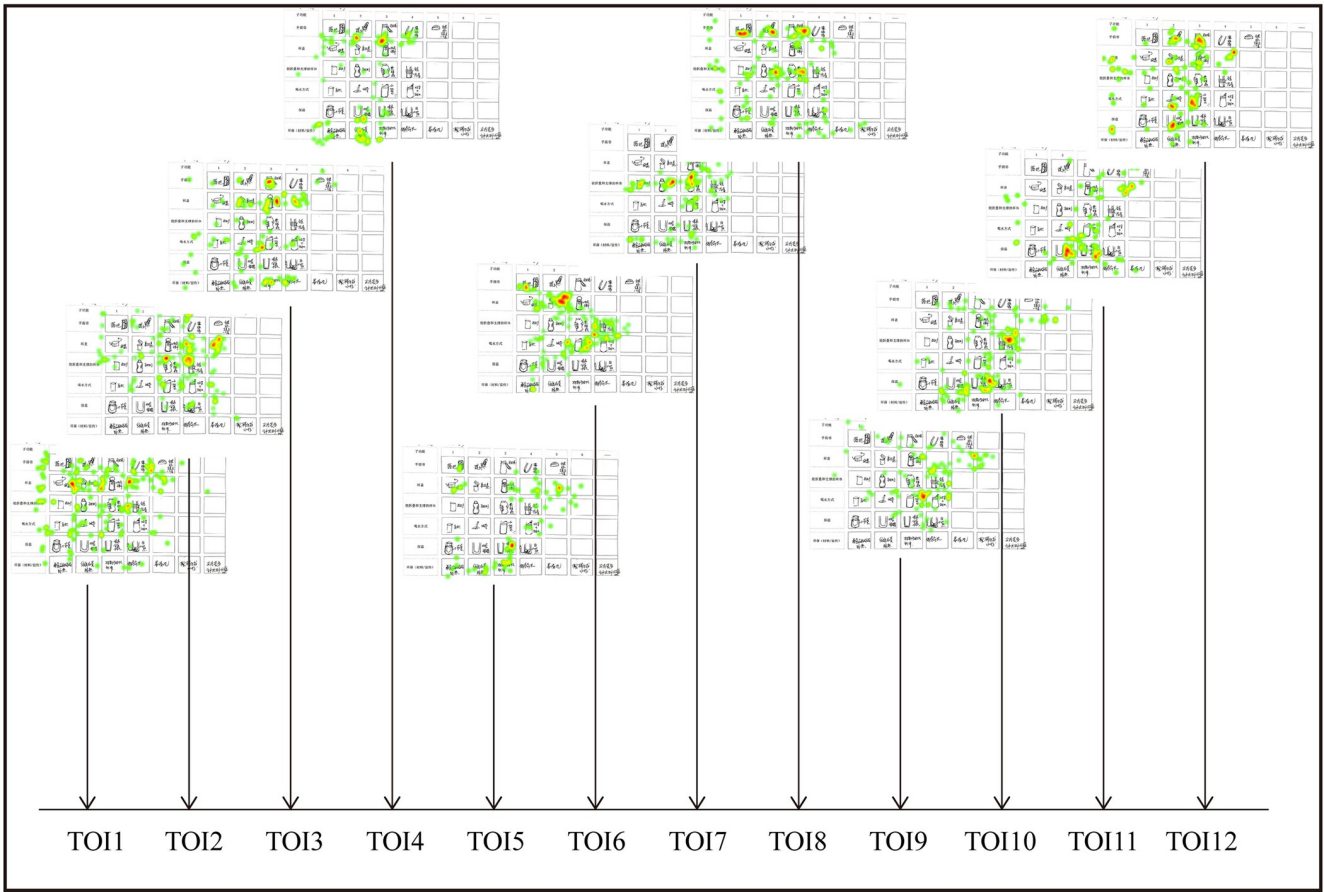
Heatmaps of the eye-tracking during 12 TOIs (an expert).

Therefore, the differences of fixation features between experts and novices can be observed preliminarily. As shown in Figs 5 and 6, novices are more likely to move in a local area while experts tend to move globally during the process of recognizing objects in different thinking modes.

#### Create areas of interest (AOI)

**Fig 7:**
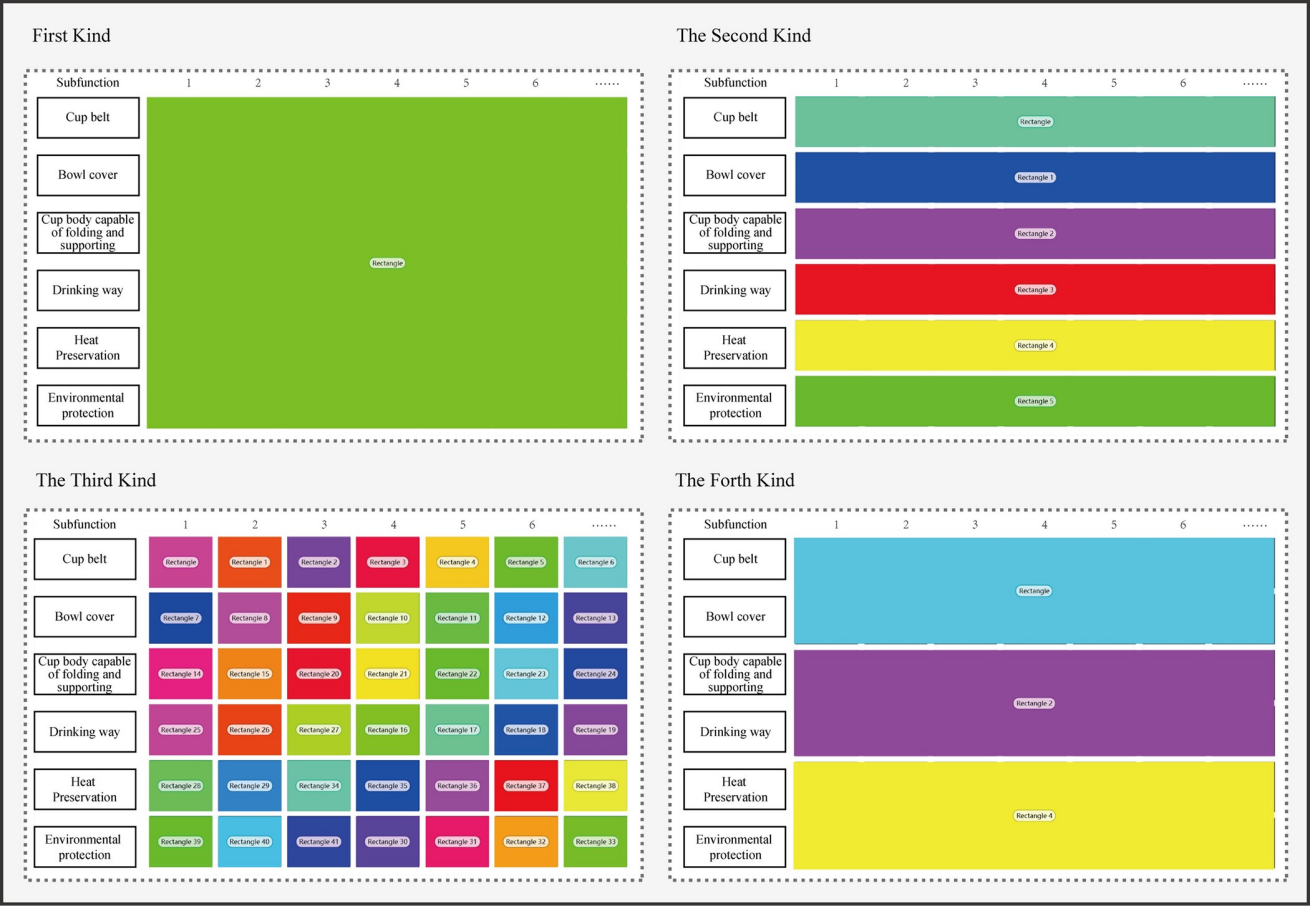
Classification of different types of AOI (area of interest).

Besides using TOI to limit the range of valid data, areas of interest (AOI) were also divided to exclude the fixation points of invalid areas. AOIs were defined as polygon areas, which contain objects of potential interest from participants. According to the metric of AOIs, we can know which area individuals first watched, how long they watched it, and whether they focused on one area or moved to another area. In this part, four different types of AOIs will be divided: i) The whole table was taken as an area of interest in order to count the subjects’ fixation duration and fixation numbers in each TOI. ii) Each sub-function line was taken as an area of interest to explore the distribution of subjects’ fixation points in each TOI. iii) Each solution was chosen as an area of interest. iv) Three blocks with different importance, defined as key, important and secondary, were taken as the area of interest. As is shown in Fig 7.

### Design scheme evaluation

**Fig 8:**
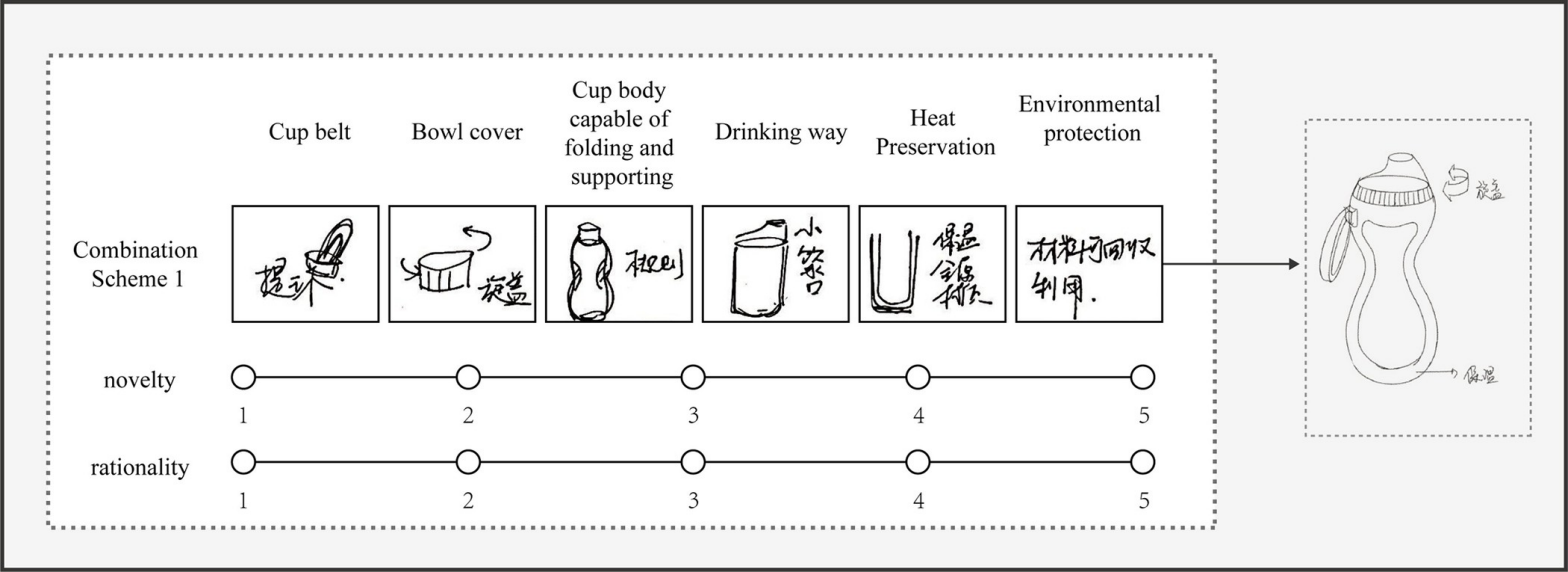
Evaluation of the second scheme of No.4 participant.

According to the research content, three indicators of rationality, innovation and quantity were selected to evaluate their final plan. Teaching assistants in design major (N = 25) were invited to use the 5-point Likert scale to score each combined scheme. For example, when observing the second plan of No.4 participant, they should consider the solution of each sub-function and the final sketch to select a score of 1–5 from innovation and rationality. As shown in Fig 8.

## Results

All participants completed the tasks within 45 minutes (M = 39.57). The number of combination schemes produced by experts (N = 12, M = 1.75, SD = 0.84) is less than that of novice (N = 12, M = 3.17, SD = 0.75), P = 0.00 < 0.05. The scores of novelty and rationality were tested between groups by independent sample T test and the reliability analysis. Results explained the reliability Alpha coefficient was 0.92, which indicated the result is credible. Besides, experts (M = 3.27, SD = 0.90) showed more novelty than novice (M = 1.77, SD = 0.21), and experts (M = 3.37, SD = 0.58) similarly showed more rationality than novices (M = 1.94, SD = 0.26). T test indicated significant difference among both variances (p = 0.00<0.05).

### Features in coverage of fixation points

First of all, the whole table was taken as AOI (area of interest). From eye tracking data, the average fixation duration with 12 TOI in expert group was 70.72, with a range of 50.83 to 94.00 sec. Note that the average fixation duration in novice group was only 35.15, with a range of 26.17 to 47.67 sec, indicating that a lower consideration occurred in novices during task process. And the T test showed that it indeed significantly differ among experts and novices group (p = 0.00<0.05).

Secondly, each solution was adjusted to be AOI. The coverage of designers’ attention in thinking activities was predicted primarily by numbers of AOI visits. The results showed that the number of AOI visits in expert group (M = 12.52, SD = 2.25, Min = 9.00, Max = 16.58) is more than those in novice group (M = 6.89, SD = 0.92, Min = 5.75, Max = 8.42), with significant difference P = 0.00. In the further analysis of its feature with the scope, each sub-functional line was defined as AOI and the AOI visits were counted. The results showed that the number of AOI visits in expert group (M = 4.59, SD = 0.48, Min = 3.67, Max = 5.42) is also higher than that in novice group (M = 2.72, SD = 0.46, Min = 2.25, Max = 3.58), with significant difference P = 0.00. In general, all these data indicated that experts have a wide range of longitudinal fixation points, with an average fixation of 4.60 sub-functional lines in a TOI, while novices are limited, with an average fixation of 2.72 sub-functional lines in a TOI. The specific values are shown in Table 3.

**Table 3:** Independent sample T test of two groups of different indicators.

index	AOI division	classify	Sample	Mean value	SD	t
Average fixation duration	The whole table	novice	12	35.15	6.16	-7.10
		expert	12	70.71	16.19	
AOI visits	Each solution	novice	12	6.89	0.91	-8.00
		expert	12	12.52	2.25	
AOI visits	Each subfunction line	novice	12	2.72	0.46	-9.69
		expert	12	4.59	0.48	

Based on the above data, the relationship between eye movements and rationality of the scheme was analyzed by SPSS software. the equation of regression analysis is Y = 0.09 X_1_ + 0.63 X_2_ + 0.625 (X_1_ = fixation duration, X_2_ = the number of AOI visits), R^2^ = 0.52, p < 0.05. Their positive correlation indicated that rationality of the scheme was promoted primarily by the number of AOI visits, somewhat less by fixation duration. These results suggested that in the process of thinking activities, the longer fixation duration and the wider range of AOI visits are more beneficial to the rationality of scheme.

### Features in the saccade amplitude of fixation points

In this process, the saccade amplitude of fixation points was divided into three levels. Saccade appearing in the same block was defined as level 1, that in two adjacent blocks was level 2 (such as jumping from block1 to block2, block2 to block1, block2 to block3, block3 to block2), and that in two blocks apart was level 3 (such as jumping from block1 to block3, block3 to block1). Within raw data AOI hit, analysis focused upon which blocks were visited and in what order visits occurred, and the number of saccades at each level were counted.

A 2-factor T test, treating experts and novices as sample, indicated that the number of saccades at level 1 was not significantly differ (P = 0.09 > 0.05). With regard to two-level-jumping, the number of saccades in expert group (M = 132.58, SD = 39.72) was higher than that in novice group (M = 42.17, SD = 18.08), and the number did indeed significantly differ (p = 0.02 < 0.05). Similarly, experts (M = 6.83, SD = 2.92) had significantly more three-level-jumping than novices (M = 0.83, SD = 0.84), which is a significant difference (P = 0.00 < 0.05). The results showed that experts are more likely to have a larger span of fixation points.

Experts made more numbers of saccades at level 1, 2 and 3 than novices, the difference between participants at level 2 and level 3 is significant. A 3-factor Regression Analysis, treating the number of saccades at level 1, 2, 3 as factors, indicated that novelty of the scheme was promoted primarily by saccades at level 3, somewhat less by saccades at level 2, and little by saccades at level 1. The regression equation is Y = 0.103 X_1_ + 0.452 X_2_ + 0.613 X_3_ + 1.08 (X_1_ = the number of saccades at level 1, X_2_ = the number of saccades at level 2, X_3_ = the number of saccades at level 3), R^2^ = 0.71, p <0,05. A decreasing trend of influencing degree was observed, this could have been a new rule of attention feature: the greater amplitude of saccades was more beneficial to the novelty of scheme.

### Features in the distribution of fixation points

According to the several groups of Heat map and Gaze plot, it was found that the attention distributions are differ in different importance areas among experts and novices. Three sub-functional blocks with different degrees of importance, treated as key, important and secondary, were taken as AOI (areas of interest) in the present analysis, in order to investigate participants’ focuses and emphasis while facing with multiple goals.

**Fig 9:**
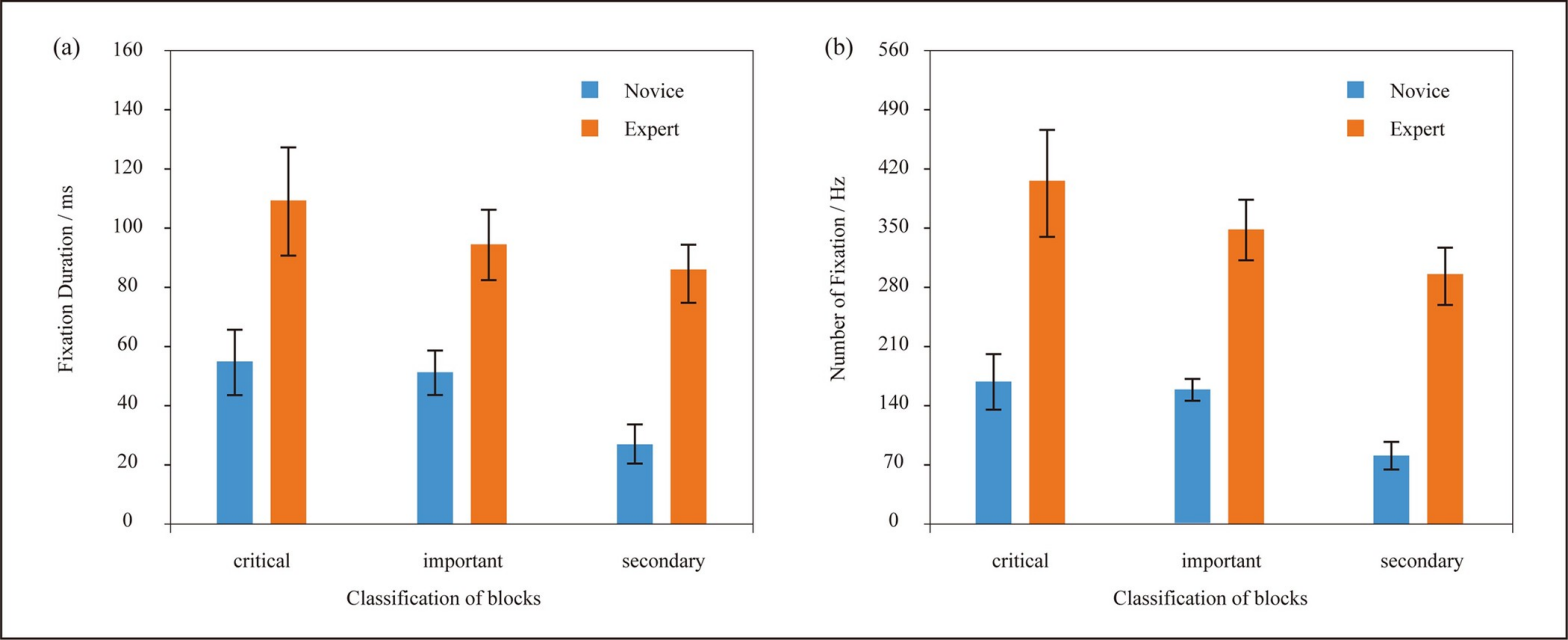
Independent sample T test of fixation duration and numbers among three blocks between groups.

A 3-factor ANOVA, treating the fixation duration in different blocks as factors, indicated that fixation duration of key block and important block didn’t have a significant difference in novice group (p = 0.7), but fixation duration of secondary block significantly differ from the other two blocks, as is shown in Table 4. As far as experts are concerned, the fixation duration had no significant difference in three blocks. Although the fixation of blocks decreases with the decrease of importance in both groups, some difference can still be found from intra-group comparison. Novices showed little fixation duration and number of fixation in secondary block, which is significantly different from those of the key and important blocks. However, those metrics of experts to the three blocks has no significant difference (Fig 9).

**Table 4:** Single factor variance analysis of fixation duration and numbers among three different blocks.

		ANOVA		LSD multiple comparison (P)			Minimum block
				Key-important	Important- secondary	Key-secondary	
novice	Fixation duration	F = 6.068	P = 0.00	0.670	0.003	0.009	secondary
	AOI visits	F = 11.475	P = 0.00	0.992	0.000	0.000	secondary
expert	Fixation duration	F = 6.726	P = 0.00	0.256	0.020	0.001	secondary
	AOI visits	F = 8.730	P = 0.00	0.339	0.005	0.000	secondary

## Discussion

The number of final combination schemes from experts is fewer than novices. As Liang found, experts are capable of controlling their cognitive processes and tend to restrict their attention to limited initial schemes, which transformed from a few core ideas [38]. However, experts showed more fixation duration, indicating that they pay more time to thinking between different goals. Previous study found that experts always spend more time than novices qualitatively analyzing, defining or framing a problem [39]. It may be because they having more professional knowledge. As Haupt said, when solving new problems, experts will recall previous knowledge and think about how to apply it to current similar problems effectively [40]. However, novices spend little time due to lacking of experience and knowledge. As well as, their focus on reasoning were decreased in design process [41]. In addition, Perkins found that the more time a designer spent on defining and understanding a problem, the better able they were to achieve a creative solution [42].

In the analysis of eye movement coverage, it was found that experts have a wider range of eye movement in thinking activities, indicating that they were more inclined to jump vertically. This may be because experts tend to move their eyes in different ways [43], and use different thinking modes during the problem-solving process. Oppositely, novices are known to use the single mode throughout the process even if it is not working [44]. Smith also pointed out that novices may ignore the context-related relationships of design problems [45], which results in a limited range of gaze point. In addition, the saccade amplitude in thinking activities of experts was also larger than that of novices, which indicates that experts are more likely to associate with knowledge in other fields when they think about solutions in one field. Moreover, analysis between saccade amplitude and novelty of the scheme shows that the weaker the correlation of association areas, the more creative ideas can be stimulated.

Finally, in order to investigate participants’ focuses and emphasis while facing with multiple goals, we divided six functional lines into key, important and secondary blocks. Results found that novices pay less attention to the secondary functions while experts can distribute their attention to blocks more evenly. Robson once insisted that only experts can extract important features and define the relationship between information blocks effectively [46], but two groups of designers in this study can both extract information of different degree. One of the reasons may be that the sub-functional lines presented by tables have certain visual analogy effect, which is beneficial for novice to distinguish blocks with different degree of importance [47].

## Conclusions

The present study collected eye movement data while individuals are thinking solutions with different thinking modes in a design task. The difference of eye movement was analyzed in several aspects, which reflecting the difference in thinking flexibility among experts and novices. The results are summarized as follows.

1. Experts showed more fixation duration in thinking activities. They tend to limit their attention to some solutions that are transformed through core ideas, so that fewer combined solutions or alternative solutions thus can be generated.
2. In terms of saccades, experts tend to think vertically among blocks while novices prefer to think horizontally within the same block. This way of thinking containing more areas is positively related to the rationality of the scheme. In addition, the thinking way of greater span is more beneficial to the novelty of scheme.
3. Designers can effectively distinguish key and unimportant blocks when facing blocks with different degree of importance, but novices tend to ignore blocks with unimportant information.

This study provides a new method for future design thinking studies and promotes the perfection and supplement of design education methodology system. It revealed the different features of eye movement in dynamic design thinking activities among experts and novices. Results will have a profound impact on the thinking development of future designers. From an educational point of view, findings highlight the role of thinking flexibility on creative design. For novices, the study provides significant features to suggest them training flexible design thinking.

Although important results have been found in this study, there are still some limitations. i) in order to control variables, the experiment has set up three sub-functional blocks for a fixed design case, so the range of saccade can only be limited to level 1–3. ii) participants in this study are concentrated on undergraduates and professors majoring in design. Because of the limited number of teachers, we have to control the number of undergraduates to control the variables. Insufficient sample may be one limitation in this study. iii) gender imbalance may also be another limitation that affects the results.

## Supporting information

S1 File Data. (XLSX)
